# Analgesia for patients undergoing shockwave lithotripsy for urinary stones – a systematic review and meta-analysis

**DOI:** 10.1590/S1677-5538.IBJU.2016.0078

**Published:** 2017

**Authors:** Omar M. Aboumarzouk, Rami Hasan, Ali Tasleem, Martin Mariappan, Rachael Hutton, John Fitzpatrick, Laura Beatty, E Jones Gareth, Tarik Amer

**Affiliations:** 1 North Bristol NHS Trust, United Kingdom;; 2 NHS Greater Glasgow and Clyde, United Kingdom;; 3 Ayr University Hospital, United Kingdom

**Keywords:** Calculi, Lithotripsy, Urinary Tract

## Abstract

**Background:**

Shock wave lithotripsy (SWL) is the first line treatment modality for a significant proportion of patients with upper urinary tracts stones. Simple analgesics, opioids and non-steroidal anti-inflammatory drugs (NSAIDs) are all suitable agents but the relative efficacy and tolerability of these agents is uncertain.

**Objectives:**

To determine the efficacy of the different types of analgesics used for the control of pain during SWL for urinary stones.

**Materials and Methods:**

We searched the Cochrane Renal Group’s Specialised Register, MEDLINE, EMBASE and also hand-searched reference lists of relevant articles (Figure-1). Randomised controlled trials (RCT’s) comparing the use of any opioid, simple analgesic or NSAID during SWL were included. These were compared with themselves, each-other or placebo. We included any route or form of administration (bolus, PCA). We excluded agents that were used for their sedative qualities. Data were extracted and assessed for quality independently by three reviewers. Meta-analyses have been performed where possible. When not possible, descriptive analyses of variables were performed. Dichotomous outcomes are reported as relative risk (RR) and measurements on continuous scales are reported as weighted mean differences (WMD) with 95% confidence intervals.

**Results:**

Overall, we included 9 RCTs (539 participants from 6 countries). Trial agents included 7 types of NSAIDs, 1 simple analgesic and 4 types of opioids. There were no significant differences in clinical efficacy or tolerability between a simple analgesic (paracetamol) and an NSAID (lornoxicam). When comparing the same simple analgesic with an opioid (tramadol), both agents provided safe and effective analgesia for the purpose of SWL with no significant differences. There were no significant differences in pain scores between NSAIDs or opioids in three studies. Adequate analgesia could be achieved more often for opioids than for NSAIDs (RR 0.358; 95% CI 043 to 0.77, P=0.0002) but consumed doses of rescue analgesia were similar between NSAIDs and opioids in two studies (P=0.58, >0.05). In terms of tolerability, there is no difference in post-operative nausea and vomiting (PONV) between the groups (RR 0.72, 95% CI 0.24 to 2.17, P=0.55). One study compared outcomes between two types of NSAIDs (diclofenac versus dexketoprofen). There were no significant differences in any of our pre-defined outcomes measures.

**Conclusion:**

Simple analgesics, NSAIDs and opioids can all reduce the pain associated with shock wave lithotripsy to a level where the procedure is tolerated. Whilst there are no compelling differences in safety or efficacy of simple analgesics and NSAIDs, analgesia is described as adequate more often for opioids than NSAIDs.

## INTRODUCTION

Urolithiasis (formation of urinary tract calculi) is common and the incidence is increasing worldwide (1). The lifetime risk is around 13% in men and 7% in women with the peak incidence in the third-to-fourth decades of life (2). Although most individuals will experience only a single episode throughout their lifetime, approximately 25% will have recurrent stone (calculi) formation (1). The process of urolithiasis occurs when urine becomes a supersaturated solution; urinary substances that are normally present in stable levels exceed the level at which they are soluble. This subsequently leads to the formation of crystals through the process of nucleation which then aggregate to form stones (3). Most commonly, stones contain calcium (calcium oxalate and calcium phosphate) with a prevalence of around 84% (1). Other types of stones include: uric acid stones (7-12%), infection (struvite) stones, (4-11%), cystine stones (<1%) and rare stone types (xanthine, 2, 8-dihydroxyadenine, indinavir) (1, 4, 5).

The aim of shock wave lithotripsy (SWL) is to cause fragmentation of a kidney stone thereby facilitating its removal or expulsion. This is achieved by targeting the stone with an externally generated shock wave that is able to propagate through the body (5). Since its introduction in the early 1980s, SWL has revolutionised the management of urinary tract stones (6). Although more than 90% of stones may be considered suitable for treatment with SWL (7, 8) success is dependent on a combination of the following factors:

- size, location and composition of stones
- patient body habitus
- performance of SWL (4)

Success rates of SWL are reported to be 50-80% but this is dependent on the factors above (5). It is important to consider that residual or larger non-fragmented stones can remain that may require either further SWL sessions or an alternative procedure, such as ureteroscopy (URS). The European Association of Urology (4) and the American Urological Association (AUA) (9) consider both SWL and URS as reasonable options for any stone that requires intervention. SWL is regarded as first line management for stones <20mm within the renal pelvis and upper or middle calices (4). It is considered the second line treatment for stones >20mm or for lower pole stones which are <20mm but have unfavourable characteristics for SWL success (shockwave-resistant stones, steep infundibular-pelvic angle, lower pole calyx >10mm, infundibulum <5mm) (4). Although SWL is less favoured for the treatment of ureteric stones, several studies have demonstrated a higher stone-free rate for proximal ureteric stones <10mm when compared to URS (4).

The energy generated by shock waves from SWL produces and influences pain in a number of ways (10).

Direct effect of shock waves on cutaneous pain receptors

Tension within the renal capsule

Movement of stone fragments

Shock wave impact to bones (11th/12th rib, transverse processes, vertebrae) and other skeletal structures

Instrumentation factors (type of lithotriptor, frequency, voltage)

Patient factors (sex, age, pain tolerance) (5, 10)

Pain relief during SWL is important, not only in providing patient comfort, but also in facilitating the success of treatment; stone targeting is improved by reducing pain-induced movements and excessive respiratory excursions (4, 10, 11). A substantial body of evidence exists that compares pain relief modalities during SWL. These include topical preparations, transcutaneous electrical nerve stimulation, anaesthetic injections (local, epidural, extradural), intravenous sedation (propofol), inhaled agents (nitrous oxide) and non-traditional methods (music, acupuncture) (10-17). Although a consensus has yet to be reached regarding the optimal pain management for patients undergoing SWL, the development of newer lithotripters that require lower energy levels and less skin surface contact has led to improvements in pain levels and consequently the need for peri-procedural analgesia (6, 10, 18).

SWL is commonly performed and is recommended as the first line treatment for a significant proportion of kidney stones (4). Given that the level of patient comfort can directly influence treatment outcome, it is essential that adequate analgesia is provided during SWL. As primarily an outpatient procedure, the use of anaesthetics and sedatives are actively discouraged (18, 19) and therefore an effective analgesic arsenal is important. A number of studies have reported the use of paracetamol (para-acetyl aminophenol), non-steroidal anti-inflammatory drugs (NSAIDs), and opioids in SWL with varying degrees of analgesic success (18, 20, 21). Therefore, debate surrounding the most effective analgesic class still remains. The current EAU guidelines recommend the use of NSAIDs for acute renal colic but do not offer advice on specific analgesics to manage pain during SWL (4). Given the lack of current consensus in the face of a relative abundance of studies on this topic, it would seem logical that a systematic review should be carried out to establish the efficacy of different analgesics for SWL.

## OBJECTIVES

The primary objective of this review was to determine the relative efficacy of the different types of analgesics used for the control of pain during SWL for urinary stones (NSAIDs, opioids, simple analgesics). The secondary objective was to evaluate the safety of the various analgesics used, the need for adjuvant analgesia and SWL parameters such as shock wave energy and duration, stone size and location. We also planned to investigate complications related to the drug therapy (such as nausea, vomiting, diarrhoea, constipation, respiratory depression and desaturation.

## MATERIALS AND METHODS

### Inclusion criteria

#### Types of studies

All randomised controlled trials (RCTs) and quasi-RCTs assessing analgesia for patients undergoing SWL were included. Comparisons included simple analgesics, NSAIDs or opioids. These trials could compare the drug classes above to themselves or to a placebo.

#### Types of participants

Any adult patient undergoing shock wave lithotripsy treatment for kidney or ureteric stones.

#### Types of interventions

The interventions of interest are the analgesic efficacy and safety of the above drug classes for the purpose of SWL. We excluded a trial involving rofecoxib which was removed from the market (22). Analgesics include para-acetyl aminophenol (paracetamol); non-steroidal anti-inflammatory drugs (NSAIDs); and opioids.

We excluded agents which were used for their sedative qualities (dexmedetomidine, propofol and midazolam infusions) as these require anaesthetic input and thus would not be relevant to modern ambulatory lithotripsy services such as those in the UK.

## Outcome measures

Studies reporting any of the following primary outcome measures were eligible for inclusion:

1. Patient reported pain assessments (visual analogue scales, verbal rating scale, simple descriptive scales) AND/OR requirement for rescue analgesia, frequency of uncontrolled pain.
2. Patient factors: age, sex, weight, height, stone burden and location.
3. Analgesic consumption (frequency and or doses).
4. Procedure variables: duration, energy, number of shocks.
5. Complications: major (renal injury, steinstrasse, bleeding and respiratory depression) and minor complications (nausea, vomiting, pain and dizziness).

### Search methods

#### Electronic searches

We searched the Cochrane Renal Group’s Specialised Register [up to 31st April 2014]. The Cochrane Renal Group’s Specialised Register contains studies identified from:

- Quarterly searches of the Cochrane Central Register of Controlled Trials CENTRAL
- Weekly searches of MEDLINE OVID SP
- Hand-searching of renal-related journals and the proceedings of major renal conferences
- Searching of the current year of EMBASE OVID SP
- Weekly current awareness alerts for selected renal journals
- Searches of the International Clinical Trials Register (ICTRP) Search Portal and ClinicalTrials.gov.

## Data collection and analysis

### Selection of studies

Medline, EMBASE and Cochrane renal databases were searched until January 2014. A combination of the following MeSH terms and keywords was used:

‘analgeisa’ or ‘shockwave lithotripsy’ or ‘NSAIDs’ or ‘opiates’, ‘simple anlagesics’ or ‘calculi’ and ‘stones’, ‘nephrolithiasis’, ‘randomised control trial’. DARE (Database of Abstracts of Reviews of Effectiveness) databases were also checked for any systematic reviews. The only language restrictions were that at least the abstract had to be in English, thus permitting extraction of relevant data. References from selected articles and reviews were also evaluated to minimise the risk of missing relevant articles.

Three authors (O.A, L.B and T.A.) followed the above inclusion criteria to select potentially relevant articles through abstract screening. Full texts of relevant articles were retrieved and screened for inclusion. Where differences of opinion emerged between the researchers regarding article eligibility, correspondence was conducted until a consensus was reached.

### Data extraction and management

Data extraction was carried out independently by three authors (T.A, O.A, R.H) and findings tabulated into MS Excel. Where more than one publication of one study existed, reports were be grouped together and only the publication with the most complete data was used in the analyses. Where relevant outcomes were only published in earlier versions, these data was used. Any discrepancy between published versions was highlighted.

### Assessment of risk of bias in included studies

The following items were independently assessed by the three authors using the risk of bias assessment tool (23).

Factors influencing bias to be assessed in the review include:

Sequence generation: Was the allocation sequence adequately generated (selection bias)?

Allocation sequence concealment: Was allocation adequately concealed (selection bias)?

Blinding: Was knowledge of the allocated intervention adequately prevented during the study (detection bias)?

Incomplete outcome data: Were incomplete outcome data adequately addressed (attrition bias)?

Selective outcome reporting: Are reports of the study free of suggestion of selective outcome reporting (reporting bias)?

Other potential sources of bias: Was the study apparently free of other problems that could put it at a high risk of bias?

### Measures of treatment effect

For dichotomous outcomes, such as pain relief assessment, patient or operator satisfaction, and duration of treatment, results will be expressed as risk ratio (RR) with 95% confidence intervals (CI). Where continuous scales of measurement are used to assess the effects of treatment, such as number of treatments required, shock waves needed, and size and location of the stones in the renal system, the mean difference (MD) will be used, or the standardised mean difference (SMD) if different scales have been used. Heterogeneity was analysed using a Chi^2^ test on N-1 degrees of freedom, with an alpha of 0.05 used for statistical significance and with the I^2^ test (24). I^2^ values of 25%, 50% and 75% correspond to low, medium and high levels of heterogeneity.

### Assessment of reporting biases

If possible, funnel plots will be used to assess for the potential existence of small study bias (23).

## Data synthesis

Data will be pooled using the random-effects model but the fixed-effect model will also be used to ensure robustness of the model chosen and susceptibility to outliers.

## Subgroup analysis and investigation of heterogeneity

Subgroup analysis will be used to explore possible sources of heterogeneity (e.g. participants, study quality, that is, analyses of the impact of studies with poor methodology on the final result, or intervention such as different lithotripters). Heterogeneity among participants could be related to age and renal pathology (stone size, location, or composition of stone). Heterogeneity in treatments could be related to prior agents used and the agent, dose and duration of therapy (different dosages of the same medication or different route of administration; or patients were on analgesics for the management of other sources of pain; or the SWL session was considered to be complicated or uncomplicated and required higher dosages of analgesia; lithotripsy differences including type and power setting used). Adverse effects will be tabulated and assessed with descriptive techniques, because they are likely to be different for various agents used. Where possible, the risk difference with 95% CI will be calculated for each adverse effect, either compared with no treatment or to another agent.

## RESULTS

### Description of studies

#### Results of the search

**Figure 1:**
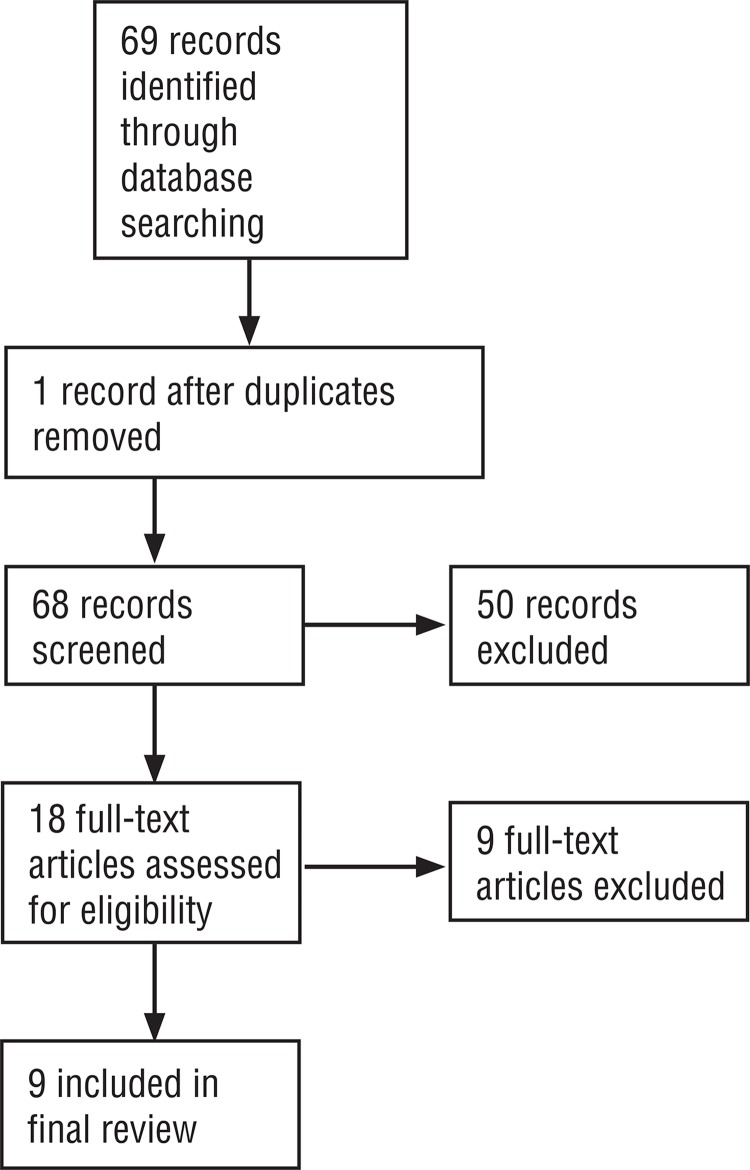
Search method for suitable studies.

The search strategy identified 68 potentially relevant citations. Of these, 50 trials were excluded on abstract review because they did not meet the inclusion criteria above. Exclusion reasons included studies not being randomized or not comparing simple analgesics, NSAIDs or opioids with themselves or placebo. We assessed 18 full text articles. 10 of these articles were excluded as described below (Figure-1).

## Included studies

Our assessment identified 9 studies that involved 539 participants (from 6 countries) which were available for inclusion dating from years 1992 to 2010 (25-33). One of these studies had multiple treatment arms (25). Trial agents included seven types of NSAIDs (lornoxicam, piroxicam, diclofeanc, keterolac, parecoxib, indomethacin and dexketoprofen), one simple analgesic (paracetamol) and five types of opioids (tramadol, remifentanil, fentanyl and morphine and pethidine). NSAIDs were compared against placebo in three studies (26, 29, 32). There were no studies comparing opioids to placebo. Two of the 9 included studies did not report variance data in a form appropriate for meta-analysis. We contacted one of the authors who replied saying that they no longer had their original data (31). We did not hear back from the second author (30). All agents were given parenterally (IM/IV) with one given orally. No studies reported major complications. Owing to the heterogeneous methods of measuring and reporting outcomes between papers, we have been unable to aggregate all outcomes in a form suitable for meta-analysis. In such instances where this occurs, we have systematically reviewed the findings and present them accordingly.

## Excluded studies

Four studies were excluded as they did not meet our inclusion criteria (epidural analgesia (14), intrathecal Sufentanil (34), accupuncutre (17, 35). Seven studies were excluded due to crossover between experimental and control arms (21, 36-40). It was possible to include one study where cross-over was present by using first phase data in this review (28). One study (22) was excluded as the trial agent, rofecoxib was excluded from the market. Another study was excluded because on close inspection of its methodology it was apparent that it was not a controlled trial (41).

We excluded 7 studies owing to the challenge of comparing analgesic efficacy in the presence of pre-medication with different agents to the study agents. As the time between pre-medicating and study drug administration was short in all studies, it was not permissive of drug washout. We therefore excluded studies where pre-medication was used in the interests of genuinely testing the clinical efficacy of trial agents. Although this was not a defined exclusion criterion, this was felt nevertheless to be a necessary step. As a result, the following seven studies were excluded (18, 20, 42-46). In the same vain, we also excluded a study where background patient controlled analgesia (PCA) in addition to the study agent was used therefore hindering direct comparisons between two agents. The manuscripts of two potentially relevant studies by Chia (43) and Yang (46) were not available. However, a recent meta-analysis by Mezentev and colleagues (6) utilising these papers enabled some degree of inspection; Yang (46) included multiple agents in research arms making it ineligible for inclusion. The full manuscript for Chia (43) was requested but was not available. A paper by Parkin (21) was also used in the same meta-analysis. We have already excluded this paper for the reasons of crossover of diclofenac in both NSAID and opioid arms.

## Risk of bias in included studies

Assessment for risk of bias is described below. Overall, the quality of included studies is low primarily due to non-robust randomisation methods and double-blinding occurring in less than half of studies. Blinding of outcome assessment is clearly defined in only three of nine studies.

## Allocation (selection bias)

There is a high risk of selection bias as only two studies (25, 27) mention a robust method of random sequence generation with the remainder not mentioning in sufficient detail to assess methodological quality in this domain. Regarding allocation, only one trial (30) described a method of randomisation which gave sufficient allocation concealment where central allocation was used. The remaining studies gave insufficient information to determine if allocation concealment was sufficient.

## Blinding (performance bias and detection bias)

In four studies (25, 30, 32, 33), participants and personnel were blinded to reduce performance bias. In three studies (26, 27, 29), participants alone were blinded. In the remaining two studies, there was no mention of blinding whatsoever (28, 31).

## Incomplete outcome data (attrition bias)

Incomplete outcomes were noted in one only study (28). However, the authors acknowledge this, enabling some data extraction.

## Selective reporting (reporting bias)

There are no cases of selective reporting in any of the included studies.

## Other potential sources of bias

In three trials (27, 30, 33), blinding of outcome assessment is clearly defined. In one study (29), blinding definitely did not occur and in five others there is insufficient information to determine if blinding of outcome assessment did occur (25, 26, 28, 31, 32). Sources of funding were noted in only one study (32).

## Simple analgesics versus NSAIDs

Only one of the included trials compared simple analgesics with NSAIDs. Demographic parameters (age, sex, weight and height) were similar between the simple analgesic group (paracetamol 1g IV) versus the NSAID group (8mg IV lornoxicam) (25). The mean stone size was 13±2.2mm and an overall stone free rate was 64.8%. Unfortunately, the authors have not divided these elements across study groups and therefore subgroup analysis in these domains cannot be performed. Pain was measured with a visual analogue scale (VAS) in this single study at enrolment and at intervals up to 30 minutes. At enrolment, pain scores were similar. Pain scores were similar between paracetamol and lornoxicam at 1, 5, 10, 15 and 25 minutes. At 20 minutes however, pain scores were significantly lower in patients taking paracetamol than those on lornoxicam (P=0.003). The post-operative pain assessment was also similar between study groups (P=0.31). There was no difference observed in the amount of supplementary analgesia required between the groups (P=0.86). Procedure duration was similar between the groups (P=0.75).

## Pain control achieved and requirement for rescue analgesia

Supplemental analgesia was administered in 21/30 and 22/30 patients in the paracetamol and lornoxicam groups respectively. This figure can be used to calculate a surrogate for “adequate analgesia” as it implies the study agents alone were not enough to achieve adequate analgesia in those participants. Therefore, 9/30 paracetamol patients and 8/30 Lornoxicam patients achieved adequate analgesia (P=0.77). There was no statistically significant difference in the total dose of supplementary Alfentanil between the two study populations (P=0.86). In two paracetamol patients and three lornoxicam patients the pain was not controlled despite PCA Alfentanil but this difference is not significant (P=0.64). The authors document that overall satisfaction about the efficacy of applied analgesia reported separately by the urologist and the patient was similar in these three groups (P>0.05).

## Adverse effects

There was no statistically significant difference in post-operative nausea and vomiting (PONV) between the groups, with 2/30 in the paracetamol arm and 3/30 in the lornoxicam arm (P=64). The mean voltage achieved was similar between the groups (P=0.30).

## Simple analgesics versus opioids

This comparison is based on the only included paper to compare the above analgesic classes. The trial by Akcali et al. (25) had three arms, the first two discussed above. Here we assess the comparison between IV paracetamol and IV tramadol. There were no significant differences in demographic variables between study groups. Once again, one cannot perform subgroup analysis regarding stone free rates or stone burdens as the authors have provided overall figures for the whole study population.

Both agents provided effective analgesia for the purpose of SWL with no significant differences apart from pain being lower in the paracetamol arm at 1 minute (P=0.03) and at 20 minutes (P=0.05). There was no difference between the two in terms of procedure duration (P=0.37).

There was more rescue analgesia required in the opioid group versus simple analgesic group but this was not statistically significant (P=0.40). The number of people who still had uncontrolled pain despite said rescue analgesia, was 3 in the simple analgesic group and 7 in opioid group but this is not statistically different (P=0.19). There was no statistically significant difference between groups in terms of mean voltage achieved (P=0.95).

## Adverse effects

There was no statistically significant difference between the two groups in terms of PONV (P=0.56) and there were no major complications reported in either group.

## NSAIDs versus opioids

Four studies including 221 patients were included in this comparison (25, 28, 30, 31).

Groups were matched for age, sex and height but not weight; with there being lighter patients in the opioid group (MD 4.87; 95% CI 1.77 to 7.97; P=0.002).

## Pre procedure baseline score

There was no difference in pre-procedure baseline VAS in the study by Ackal, the only team to assess this in this comparison (25).

## Patient rated pain scores

**Figure 2:**
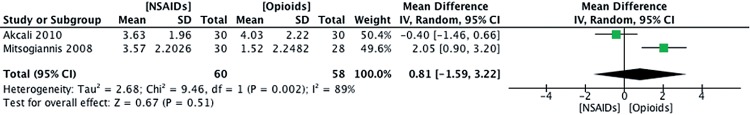
Comparison between NSAIDs and opioids, visual analogue score at 10 minutes.

**Figure 3:**
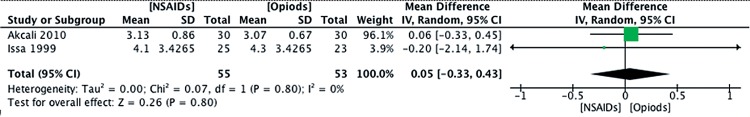
Comparison between NSAIDS and opioids, post-op visual analogue score.

There was no statistically significant difference in pain scores at 1, 5, 10 or 15 minutes in two studies (25, 30). This was also the case post-operatively at 30 minutes (P=0.80) (Figure-3). At 10 minutes, lower VAS scores were recorded in the opioid groups (25, 31) but on meta-analysis this is not significant (Figure-2: MD: 0.81, 95%CI-1.59 to 3.22; P=0.51). Mitsogiannis did not provide variance data and, as such for purposes of meta-analysis this was extrapolated from the provided P value in a method described by the Cochrane handbook (P<0.001). The author was contacted in order to obtain the original data. Unfortunately, this data is no longer available. In the same study, the mean pain score in patients in the NSAID group, after the first dose of analgesia, was significantly higher than patients in the opioid group (3.57 versus 1.76, respectively (P<0.001). However, in patients who responded to the first dose of analgesia, the mean pain scores were similar (P=0.20). After administration of supplementary analgesia, the mean pain scores reported by the patients in both groups did not differ significantly (mean 1.56 versus 1.82, respectively, P=0.21) (31).

## Supplementary analgesia

The total dose of rescue analgesia (PCA) in the one included study that reported this was lower for NSAIDs but was not statistically significant (P=0.58) (25). Issa reports no statistically significant difference between NSAIDs and opioids in terms of supplemental analgesia (P>0.05). Due to an absence of provided variance data this data cannot be pooled for meta-analysis (30).

## Adequate analgesia

**Figure 4:**
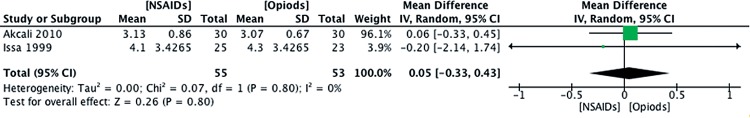
Comparison between NSAIDs and opioids, adequate analgesia achieved.

This was calculated by assessing those patients who did not require additional analgesia (either a second dose of study agent or an alternative agent for breakthrough pain). On meta-anaylsis, analgesia could be defined ‘adequate’ more often for opioids than for NSAIDs (Figure-4: RR 0.358; 95% CI 043 to 0.77, P=0.0002) (25, 28, 31).

## Uncontrolled pain despite additional analgesia

This measure was recorded in one study (25), despite there being more cases of uncontrolled pain in the opioid group this is not significant (P=0.09).

## Procedure duration

There was no difference in procedure duration between NSAIDs and opioids in terms of procedure duration (MD 0.67, 95% CI from -0.24 to 1.58, P=0.15) (25).

## Complications

There was no statistically significant difference between three of the four studies (25, 30, 31).

## Voltage

There was no statistically significant difference in mean voltage achieved between NSAIDs and opioids (MD 0.80, 95% CI from -0.45 to 2.05, P=0.21) (25), or the percentage mean maximum shock wave level achieved between the groups (MD-0.40, 95% CI from -1.22 to 0.42, P=0.34) (31). Another study found no difference in the number of patients reaching a maximum energy level of 26kv, (RR 0.19, 95% CI from 0.01 to 4.05; P=0.28) (28).

## Number of shocks

There were no differences in the number of shock waves between NSAIDs and opioids (MD -15.00, 95% CI from -34.13 to 4.13, P=0.51) (31). Issa et al. (30) provided no variance data and as such their data regarding this outcome cannot be included for comparison.

## Number not completing SWL

One patient in the NSAID group (parecoxib) did not complete SWL and this was not statistically significant (RR 3.21, 95% CI from 0.14 to 75.61, P=0.47) (31).

## Respiratory depression

This was reported in two studies and there was no difference between groups (30).

## NSAIDs versus NSAIDs

One study suitable for inclusion compared outcomes between two types of NSAIDs (diclofenac versus dexketoprofen). There were no statistically significant differences with regards to age, height or weight but only males were included in this study (33). There was no statistically significant difference in proportion of renal versus ureteric stones between study groups. There were no differences in tolerable pain (RR 0.98, 95% CI from 0.81 to 1.19, P=0.84), intolerable pain (RR 2.25, 95% CI from 0.49 to 10.38, P=0.29) or stone burden (MD -1.43, 95% CI from -34.30 to 31.44, P=0.89). However, when pain was reported as intolerable the mean VAS was significantly higher in the diclofenac group versus dexketroprofen (MD 0.97, 95% CI from 0.16 to 1.78, P=0.02).

## Opioids versus Opioids

Cortinez and colleagues compared remifentanil and Fentanyl (27). There were no differences between the groups in terms of demographics or stone location. There were no differences between the groups in terms of mean shock wave energy achieved (P=1.00) or mean opioid infusion rate (MD 0.01, 95% CI from -0.03 to 0.05, P=0.59). The procedure was longer in the fentanyl group but this was not significant (MD -8.00 mins, 95% CI from -19.82 to 3.82, P=0.18). There were more cases of PONV in the fentanyl group 18 versus 3 (RR 0.17, 95% CI from 0.06 to 0.49, P=0.001) and this was significant. There was more intra-operative nausea in the fentanyl group (RR 0.05, 95% CI from 0.00 to 0.85, P=0.04). There were significantly fewer cases of intra-op (RR 0.46, 95% CI from 0.21 to 0.99, P=0.04) and post-op desaturation in the remifentanyl group than the fentanyl group (RR 0.13, 95% CI from 0.02 to 0.92, P=0.04). More patients in the Fentanyl group required supplementary analgesia during SWL but this was not significant (RR 0.11, 95% CI from 0.01 to 1.95, P=0.13). Post procedure, more patients in the remifentanyl group required additional analgesia but this is not significant (RR 7.00, 95% CI from 0.38 to 128.02, P=0.19). Patients in the remifentanyl group had a higher sedation score and this is significant (RR 0.52, 95% CI from 0.34 to 0.80, P=0.003). There were more cases of persisting pain post procedure in the remifentanyl group but this is not significant (RR 3.50, 05% CI from 0.82 to 15.01, P=0.09).

## NSAIDs versus Placebo

Three studies compared NSAIDs versus placebo. Aybek and colleagues compared 40mg IM piroxicam (26), Fredman et al. diclofenac sodium 75mg IM29 and Ou et al. indomethacin capsules 50mg commencing shortly after the procedure (32). There were no significant demographic differences between the groups (26, 29, 32) or differences in stone fragmentation rates (RR 1.62, 95% CI from 0.59 to 4.46, P=0.35) (29). The attained shock wave voltage was similar in the single study which recorded this (P=1) (26). There was no difference in procedure duration (MD-3.41; 95% CI from -8.25 to 1.43; P=0.17) (26, 29).

The verbal rating score was lower in the NSAID group (piroxicam) at all intra-operative points with P<0.05 in the one study measuring intra-op pain (26). Post operatively, pain was also lower in two studies; VRS at 6 hours was significantly less in the NSAID group (P<0.00001, MD=-0.95, 95% CI -1.07, -0.83) (26, 29) and at both 12 hours (P<0.0001) and 24 hours (P=0.0004) (26).

On meta-anaylsis, a greater number of shocks were tolerated by NSAIDs (P=0.001; MD 404.18; 95% CI 98.68 to 709.68) (26, 29). Procedure duration was shorter for NSAIDs but not significantly so (MD -3.41, 95% CI from -8.25 to 1.43, P=0.17) (26, 29).

There were more cases of intractable pain in the placebo group (RR 0.24; CI: 0.10 to 0.59; P=0.002) (26, 29). There was no increase in cases of ureteric colic after SWL between NSAID and placebo (RR 0.44, 95% CI from 0.15 to 1.29, P=0.13 (32). There was no significant difference in NSAID patients requiring pethidine for breakthrough pain (RR 0.64, 95% CI from 0.31 to 1.31, P=0.22) (26, 32). The number of patients needing additional analgesia as well as trial agents was significantly less in the one study measuring this, RR 0.25, CI from 0.08 to 0.80, P=0.02 (32). Fredman et al. found the total opioid dose for breakthrough pain was less in the NSAID group versus placebo but not significant (MD -30.00, 95% CI from -98.04 to 38.04, P=0.39). The same authors found no difference in midazolam consumption (MD -0.10, 95% CI from -1.10 to 0.90, P=0.84) or mean voltage (P=1) (29).

## DISCUSSION

This review aimed to assess the relative clinical efficacy of simple analgesics, NSAIDs or opioids during shock wave lithotripsy for renal calculi. Based on the one study comparing a simple analgesic (paracetamol) versus an NSAID (lornoxicam), there were no overall significant differences in clinical efficacy or tolerability between these agents. Only one study is included in this subgroup and therefore generalisable conclusions are limited. In summary, both paracetamol and lornoxicam are tolerated by patients to provide adequate analgesia during SWL (25). The same study (of three arms) also compared paracetamol versus tramadol during SWL (25). Once again, overall there was no significant difference in pain scores between the agents at various fixed time points over 30 minutes. No major complications were reported and there was no difference in the number of patients with PONV (P=0.56). Once again, both agents provide safe and effective analgesia for the purpose of SWL.

Overall, there was no significant difference in pain scores between NSAIDs or opioids in the three studies comparing these drug classes. At 10 minutes however, lower VAS scores were recorded in the opioid groups but on meta-analysis this was not significant (MD 0.73; 95% from -0.05 to 1.51, P=0.07) (25, 31). One limitation was that one of the included studies did not provide variance data and as such for purposes of meta-analysis this was extrapolated from the provided P value (P<0.001) using previously reported techniques. In the same study (31), the mean pain score in patients in the NSAID group, after the first dose of analgesia, was significantly higher than patients in the opioid group (3.57 versus 1.76, respectively (P<0.001). However, in patients who responded to the first dose of analgesia, the mean pain scores were similar (P=0.20). After administration of supplementary analgesia, the mean pain scores reported by the patients in both groups did not differ significantly (mean 1.56 versus 1.82, respectively, P=0.21). In terms of achieving adequate analgesia, this was calculated by assessing those patients who did not require additional analgesia (either a second dose of study agent or an alternative agent for breakthrough pain). On meta-anaylsis, analgesia could be defined as adequate more often for opioids than for NSAIDs (P=0.0001) (25, 31). However, this figure is limited by the considerable heterogeneity (I^2^=93%) between studies thus limiting generalisability. In terms of actual doses of consumed rescue analgesia, two studies found similar doses between NSAIDs and opioids (P=0.58, >0.05) (25, 30). The absence of variance data meant that data could not be pooled from one paper (30). In terms of tolerability, there is no difference in PONV between the groups (RR 0.72; 95% CI 0.24 to 2.17; P=0.55) and only one patient in the NSAID group (parecoxib) did not complete SWL (P=0.47) (31). There was no difference in number of cases of respiratory depression (P=0.26) (31). There is no statistically significant difference in mean voltage achieved (P=0.21) (25) or the percentage of maximum shock wave energy achieved (P=0.34) (25, 31).

One study suitable for inclusion compared outcomes between two types of NSAIDs (diclofenac versus dexketoprofen). There were no significant differences in any of our pre-defined outcomes measures. However, when pain was reported as ‘intolerable’ the mean VAS was higher in the diclofenac group versus dexketroprofen (P=0.02). This study builds on the meta-analysis of three studies by Mezentsev which found that there were no significant difference in efficacy between NSAIDs and opiods for SWL (6).This study builds on this by including more studies and not limiting to type of lithotripter as well as comparing more analgesic classes.

Limitations of this study include the small number of studies suitable for inclusion and the heterogeneity in the reporting of outcome measures. This hinders precise inter-article comparison. The lack of variance data in some studies meant that pooled analysis was done by extrapolating from a provided P value which has implications in terms of pooled analysis. There are numerous sources of bias as described above; notably most studies having non-robust randomisation methods and double-blinding occurring in less than half of studies. Blinding of outcome assessment was clearly defined in only one third of studies.

## CONCLUSIONS

This systematic review and meta-analysis distils the literature in this area to show that simple analgesics, opioids and NSAIDs all provide adequate analgesia for the purpose of SWL. In one paper (25), clinical efficacy and tolerability was similar between all three classes. However, against some criteria, meta-anaylsis has shown opioids to offer superior efficacy than NSAIDs. Indeed, pooled data from two studies shows that analgesia could be defined as adequate more often for opioids than for NSAIDs (P=0.0001) (25, 31). On meta-analysis of other outcomes however, there were no significant differences between groups across a range of markers of efficacy; these include consumed doses of rescue analgesia (25, 30), and the percentage of maximum shock wave energy achieved (25, 31). When comparing two opioids (fentanyl versus alfentanil) whilst there were no differences in efficacy, alfentanil was better tolerated.

Overall, NSAIDs, opioids and simple analgesics all provide adequate analgesia for the purposes of SWL. NSAIDs are of more value for SWL than placebo. One would anticipate more side effects with opioids than simple analgesics or NSAIDs. However, this study has not demonstrated this. It would be sensible on the back of this research to address analgesic requirements for SWL with a simple analgesic such as paracetamol. Breakthrough pain could be addressed with NSAIDs initially then opioids. There are an array of lithotripers used in the included studies which may impact on efficacy, pain and tolerability of the procedure. Further research is required in this area to compare the pain associated with different lithotripsy devices. Success rates for ESWL treatment have been reported to be machine-dependant with one study showing a higher stone-free rate and lower re-treatment rate with the HM3 lithotriptor (47). Economic evaluation was not reported as an outcome in any of the studies. This will become increasingly relevant to Urology departments in the future.
